# Supplementary material for: Structure and exfoliation mechanism of two-dimensional boron nanosheets
Source: Nat Commun. 2024 Jul 20;15:6122. doi: 10.1038/s41467-024-49974-8 (PMC11271264; doi:10.1038/s41467-024-49974-8)
Supplement: Supplementary file 1 — Supplementary Information [file 41467_2024_49974_MOESM1_ESM.pdf]

Supplementary Information

Structure and exfoliation mechanism of two-dimensional boron nanosheets

Jing-Yang Chung [1,2,†], Yanwen Yuan [1,2,†], Tara P. Mishra [1], Chithralekha Joseph [1],

Pieremanuele Canepa [1], Pranay Ranjan [3], El Hadi S. Sadki [4],

Silvija Gradečak *[1,2] & Slaven Garaj *[1,5,6]

[1] Department of Materials Science and Engineering, National University of Singapore, Singapore 117575

[2] Applied Materials - NUS Advanced Materials Corporate Lab, National University of Singapore, Singapore 117608

[3] Department of Metallurgical and Materials Engineering, Indian Institute of Technology Jodhpur, Jodhpur, Rajasthan-342037, India

[4] Department of Physics, College of Science, United Arab Emirates University, Al-Ain, UAE

[5] Department of Physics, Centre for Advanced 2D Materials, National University of Singapore, Singapore 117546

[6] Department of Biomedical Engineering, National University of Singapore, Singapore 117583

[†] These authors contributed equally: Jing-Yang Chung, Yanwen Yuan

* Email: gradecak@nus.edu.sg, slaven@nus.edu.sg

SUPPLEMENTARY FIGURES

Microstructural morphology of 3D boron crystals

To obtain better quantification of grain sizes, multiple polarized light microscope images were taken for each sample (higher magnification in **Supplementary Fig. 1a** and larger field-of-view in **Supplementary Fig. 1b**). Sample B shows higher defect densities than Sample A (**Supplementary Fig. 2**), while electron back-scattered diffraction image (EBSD) shows dense density of defects in Sample C, matching the trend of XRD peak-broadening in **Fig. 2f** in the main text. Maps of the grain distribution were obtained through ImageJ thresholding processing (**Supplementary Fig. 1b**). For each sample, the areas of > 300 grains were obtained, and their radii are calculated assuming a circular morphology. As shown in the histograms in **Supplementary Fig. 1c**, Samples A and B have comparable grain sizes, while Sample C is roughly a magnitude smaller.

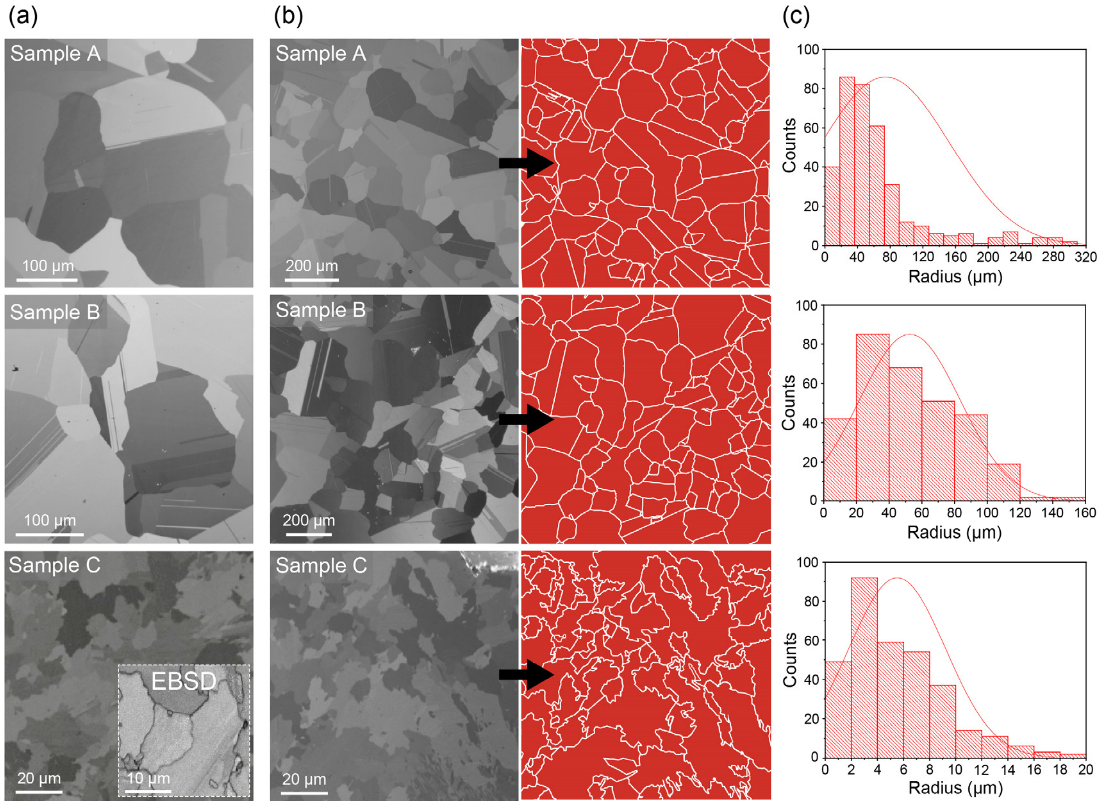

Supplementary Fig. 1. (**a**) Polarized light microscope images of Samples A, B and C, and (**b**) larger field-of-view images with corresponding maps of the grains shown on the right. (**c**) Histograms showing the grain radius distributions of Samples A, B, and C.

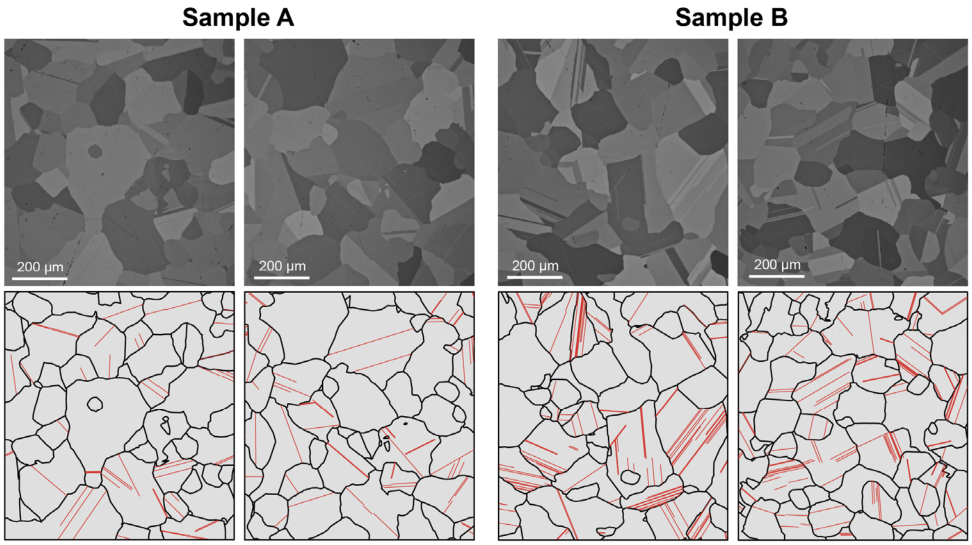

Supplementary Fig. 2. Polarized light microscope images comparing the defect density in Samples A and B. In the grain maps below the images, the defects are illustrated in red.

To further illustrate the presence of high density of planar defects in Sample C, the STEM and TEM images in **Supplementary Fig. 3** portray grains with and without planar defects. Similar to the TEM image in **Figure 5a** in the main text, the parallel streaks of strain contrast suggest that the planar defects are also parallel to each other on a large scale. TEM samples were randomly extracted from 3D boron pieces from Samples A, B, and C, as shown in the inset in **Supplementary Fig. 3a** through focused ion beam (FIB) lift-off [1].

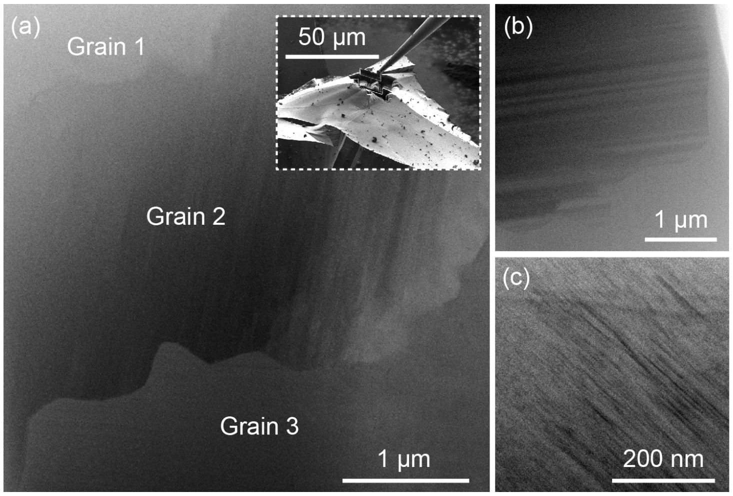

Supplementary Fig. 3. (**a**) and (**b**) LAADF STEM, and (**c**) BF TEM images demonstrating the streaks of strain-contrast, indicating defects in Sample C. The inset shows the FIB lift-out.

Distribution of liquid-phase exfoliated boron products

The color and transparency of the sonicated products dispersed in the liquid medium following liquid phase exfoliation (LPE) typically can provide insights into sheet concentration for vdW materials [2]; here, we show that for 3D boron, the light dispersion involves both 2D sheets and 3D particles. **Supplementary Fig. 4a** shows sonicated product dispersion from Samples A, B, and C to be similar to LPE boron reported in the literature. However, prior to centrifugation, **Supplementary Fig. 4b** demonstrates the distribution of sonicated products to consist of nanosized 3D particles and 2D 'flakes', which appear transparent, thin, and wedged. Cross-comparison with high-angle annular dark-field STEM shows these larger 'flakes' to be thicker than the surrounding carbon grid (see **Supplementary Figs. 4 and 5**), illustrating the importance of STEM characterization in ensuring structural characterization is not performed on thicker particles.

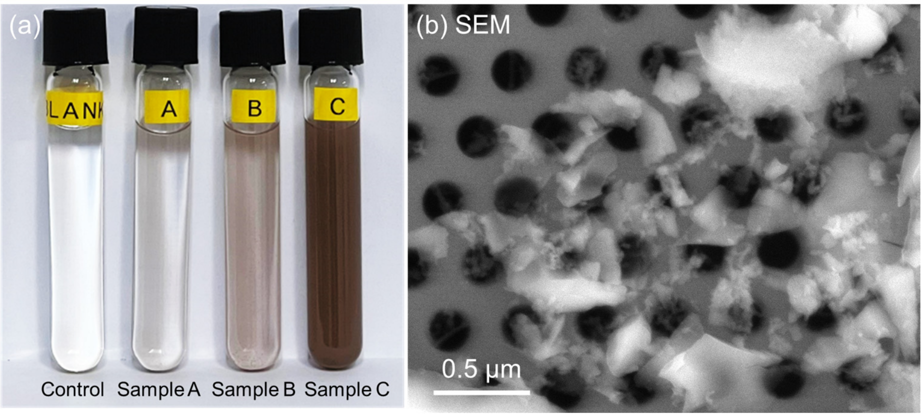

Supplementary Fig. 4. (**a**) Photographs showing the sonicated product dispersion of Samples A, B, C, and a control vial containing the sonicated solvent without boron. (**b**) SEM image of the distribution of the sonicated products.

Flake thickness through TEM and STEM

The presence or absence of Kikuchi patterns formed from thermally diffused scattered electrons in a sample provides a quick inspection of the relative sample thickness. Kikuchi patterns are apparent from a thicker sample in **Supplementary Fig. 5a** (see inset) with stronger HAADF STEM contrast than the supporting carbon film. Combined, these imaging modes ensured that only thin 2D sheets were selected for atomic-scale characterization in this study.

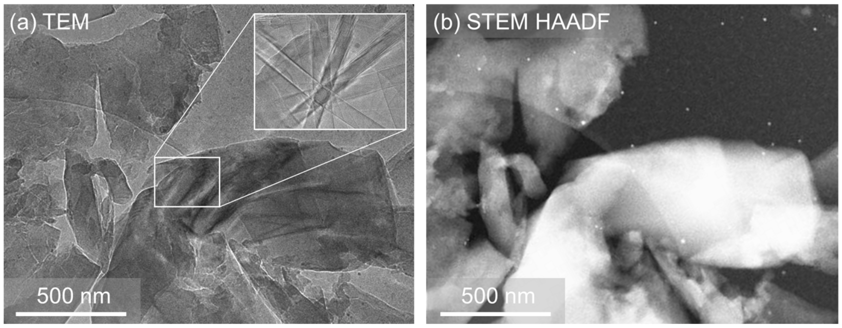

Supplementary Fig. 5. Example of a platelet suspended on a holey carbon grid imaged through (**a**) converged beam TEM imaged at the back focal plane, and corresponding (**b**) HAADF STEM. Unlike the case in **Figure 1g** in the main text, the Kikuchi pattern formed from thermally diffused scattered electrons (see inset) is evident in (a), indicating a thicker platelet.

Due to the lower sensitivity of TEM imaging in mass/thickness contrast, thicker platelets—though still electron transparent—may appear as 'thin' and 'layered.' This is exemplified by the intensity profiles across the same regions in **Supplementary Figs. 6a–b**, where the difference in contrast between the thick/thin samples is relatively insignificant in TEM, but shows high thickness-dependences in HAADF STEM. For studies employing conventional parallel beam TEM, cross-referencing to AFM or other height measurement studies is critical to ensure that the atomic-scale characterization is not performed on bulk-like platelets instead.

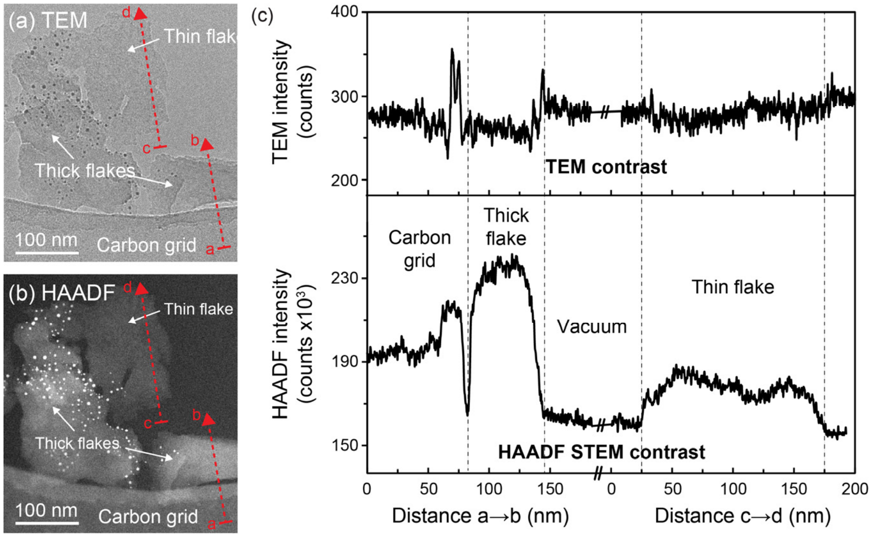

Supplementary Fig. 6. (**a**) TEM and corresponding (**b**) HAADF STEM images, taken at the same location. (**c**) The intensity line scans across the samples marked in (a) and (b) shows the intensity ratio differences between the two imaging modes.

Flake thickness through AFM

Boron flake thickness and morphology from Sample C post-centrifugation were studied through AFM. In **Supplementary Fig. 7a** (as well as the larger field-of-view image in **Supplementary Fig. 7b**), height topography images of the flakes are shown (height line scan in **Supplementary Fig. 7c** suggest the flake height to be ~ 4 nm). 100 flakes (similar to those in the representative image in **Supplementary Fig. 7b**) were analyzed, giving a mean flake area of 0.02 μm^2 and mean thickness of 3.9 nm. We note that these values are similar to those reported previously for LPE boron [3]. Distribution of the characteristic width of the flakes $\langle L \rangle$ and its dependence on the flake thickness are shown in **Supplementary Figs. 7d and e**, respectively. Characteristic width is defined as the square root of the flake area, $\langle L \rangle = A^{1/2}$. It should also be mentioned that from AFM alone, the shape of the boron flakes from LPE appears to be indistinguishable from borophene grown on metal substrates [4], and atomic-resolved characterization is required to conclusively obtain the crystal phase.

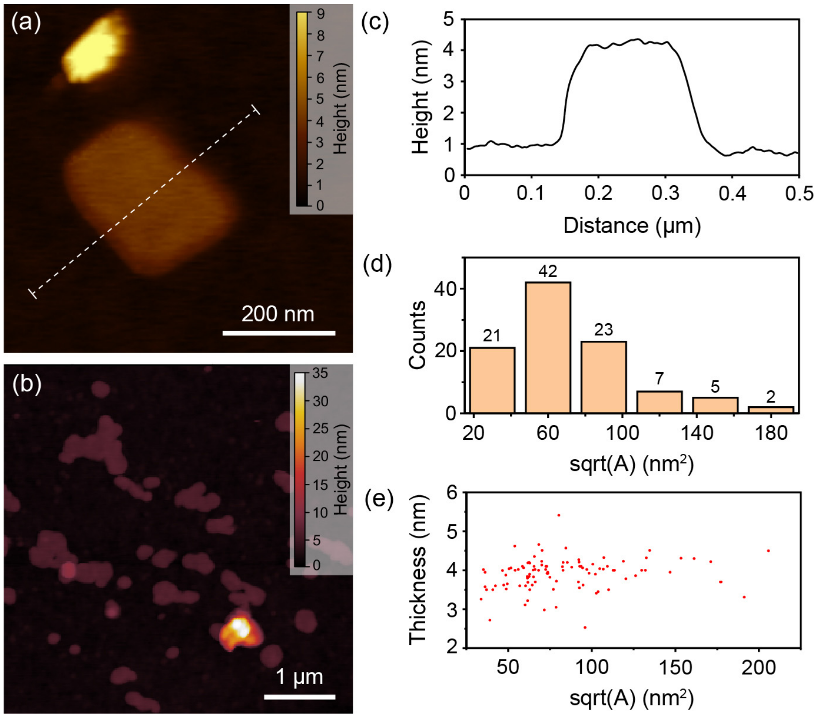

Supplementary Fig. 7. (**a**) Example AFM topography image of a single boron flake, and (**b**) large-field-of-view AFM topography image. (**c**) Corresponding height profile of the flake in (a) showing a thickness of roughly 4 nm. Multiple large-field-of-view images were captured to obtain the lateral dimension (square root of measured area) histogram in (**d**). (**e**) Scatterplot of flake thickness as a function of lateral dimension.

Observation of boron-organic complexes and surfactants

Here, we would like to draw attention that many 'flakes' seen in AFM may be contaminant by-products from sonication, rather than true boron sheets. Post-LPE, 'sheet-like' layered products with irregular shapes (**Supplementary Figs. 8a–b** and **Supplementary Figs. 9a–b**) can often be seen. However, these sheet-like products are likely organic complexes merged with the bulk material, as repeated SEM/AFM scans result in their morphology change (**Supplementary Figs. 8c–d** and **Supplementary Figs. 9c–d**), unlike for the case shown in **Supplementary Fig. 7**. Raman spectra in **Supplementary Fig. 9e** demonstrate the 'sheets' display an enhanced peak from the solvent.

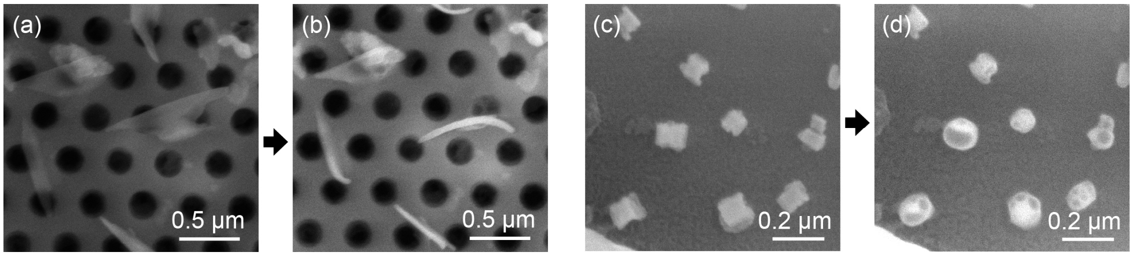

Supplementary Fig. 8. (**a**)–(**b**) SEM images showing the appearances of transparent 'sheets' and high symmetry products. (**c**)–(**d**) Their appearances after repeated SEM scans.

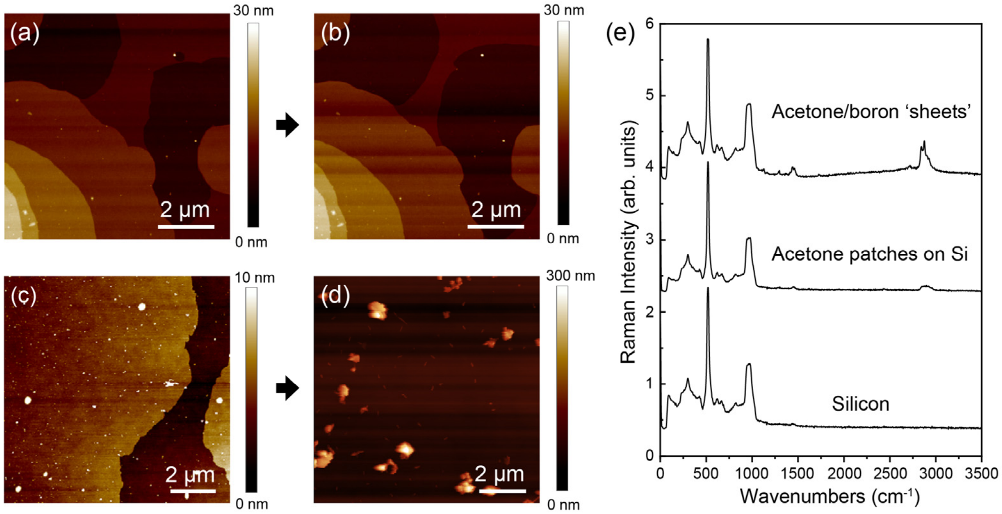

Supplementary Fig. 9. (**a**)–(**b**) Large layered-like products observed under AFM. Likewise, repeated AFM scans also result in the structural disintegration of the sheets (**c**), while high-temperature thermal annealing removes these artifacts completely (**d**). (**e**) Raman spectra of the silicon wafer, pure acetone solvent patches on silicon, and the 'layered sheets' on silicon.

Importance of STEM imaging parameters

In this section, we show examples demonstrating the importance of coherent imaging methods for characterizing icosahedral-based boron materials. As shown through the examples in **Supplementary Fig. 10**, the incoherently and semi-incoherently scattered electrons acquired with collection angles of 45–180 and 25–110 mrad do not provide sufficient contrast to distinguish the lattice arrangement of β-rhombohedral boron (viewed down the $[1\bar{1}0]$ zone-axis). Comparing between annular bright-field (15–45 mrad) and center bright-field (~0–15 mrad), we find the latter to provide the best separation between the atomic column positions, and the best intensity contrast between atoms and vacuum.

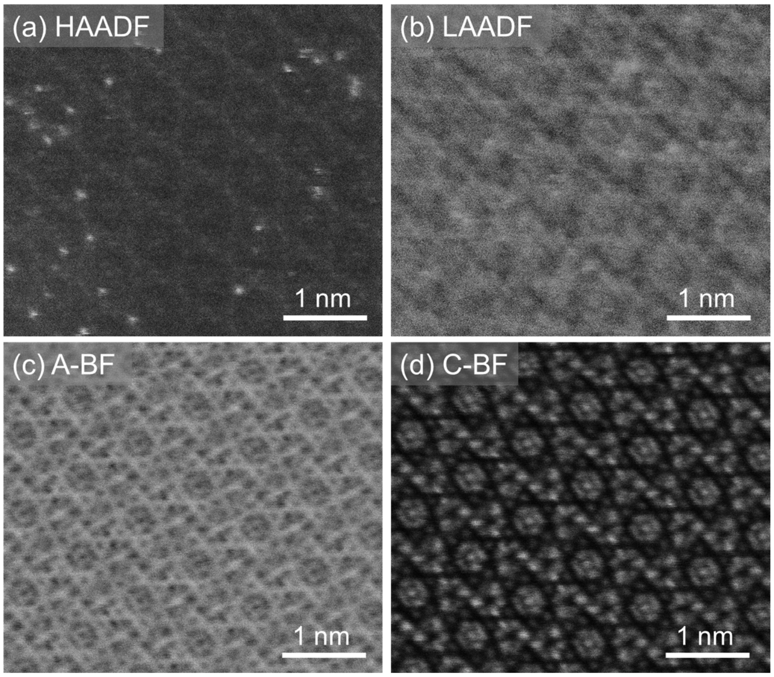

Supplementary Fig. 10. Comparison between STEM collection angles β: (**a**) HAADF, 45–180 mrad, (**b**) LAADF, 25–110 mrad, (**c**) A-BF, 15–45 mrad, and (**d**) C-BF, 0-15 mrad.

Surface passivation of boron sheets with oxygen

Similar to previous LPE reports of non-vdW materials [3,5], we show here that oxygen likely plays a key role in the surface passivation of boron sheets, stabilizing the suspension. Supporting our electron energy loss spectroscopy (EELS) elemental results presented in **Fig. 2b** in the main text, energy dispersive X-ray spectroscopy (EDS) performed in the STEM and X-ray photoelectron spectroscopy (XPS) were used to measure the oxygen distribution in the

exfoliated boron products. EDS mapping was performed on a thick platelet to contrast the higher percentage of oxygen in the thin (edge) regions of the platelet compared to that of the bulk. This demonstrates that oxygen is only present on the surfaces, rather than integrated into the crystal. XPS analysis also indicates that no gross-scale bonding changes occurred during the LPE process; previous reports have attributed the appearance of the B–O peak to the native oxide in bulk boron [6,7]. This impurity element is likely the factor leading to the bright spots in HAADF imaging on bulk boron (see **Supplementary Fig. 10a**).

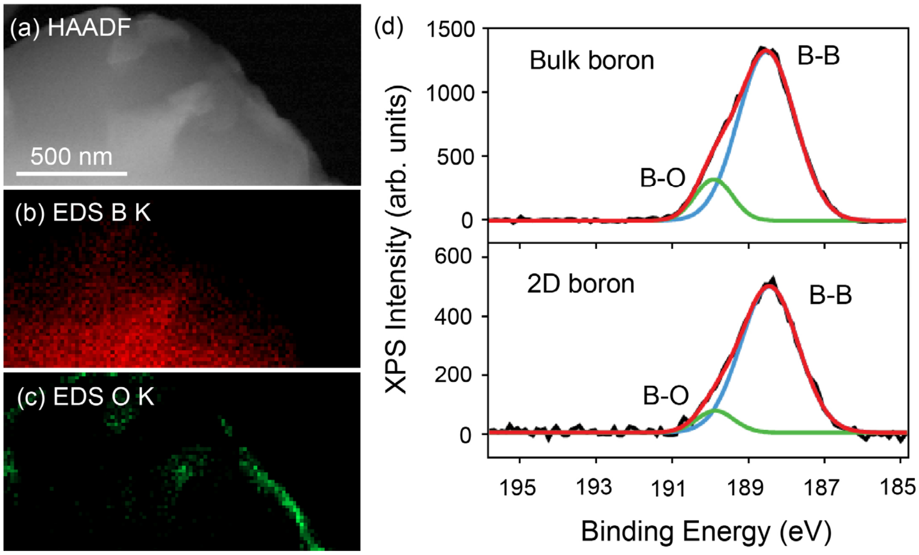

Supplementary Fig. 11. (**a**) HAADF, and corresponding EDS maps of (**b**) B and (**c**) O. Oxygen concentration is higher along the edges of the flake, which are thinner, suggesting that only the surfaces of LPE sheets are passivated by oxygen. (**d**) XPS spectrum of the exfoliated sheet.

Raman spectra of LPE boron

Supplementary Fig. 12 presents Raman data for three sonicated samples. Raman spectrum for Sample A shows minimal changes after sonication, compared to Raman data before sonication (**Fig. 2f** in the main text). In contrast, Samples B and C exhibited significant changes, indicating structural changes occurring during sonication. These results support the observation that Sample A has less propensity to break into sheets than Samples B and C. After LPE, Raman spectra of Samples B and C change significantly compared to the original values, but they remain similar to each other.

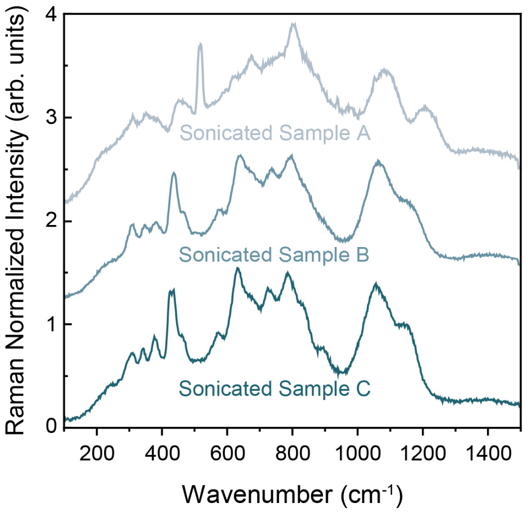

Supplementary Fig. 12. Raman spectra of exfoliated boron from Samples A, B, and C. Unlike Samples B and C, the spectrum of Sample A bears more semblance to the bulk 3D.

Influence of small angle tilts on the projected atomic images of icosahedral-based boron

As described in **Supplementary Fig. 5**, the nanosheet imaging was performed without any tilting due to the lack of Kikuchi patterns. As a result, STEM images taken on the boron nanosheets often do not appear as that of the perfectly aligned (001) plane(s) described in the main text in **Fig. 3c**. An example is shown in **Supplementary Fig. 13a**. As a result of small tilts away from the zone axis, the projected images can be oriented away from the (001) plane. Nevertheless, we show through the similarity of the simulated image in **Supplementary Fig. 13b** that such tilted projections still indicate stacked arrangements of the (001) plane.

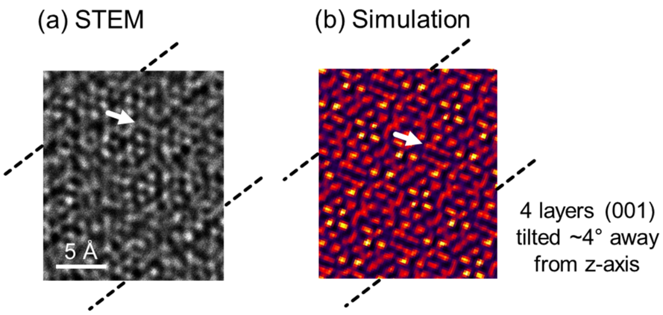

Supplementary Fig. 13. (**a**) Example experimental STEM C-BF image on the boron nanosheet taken without any intentional sample tilting. (**b**) Simulated image of 4 layers of the (001) plane, similar to that in the main text (**Fig. 3e**) but tilted ~4° away from the electron beam. The dotted lines and arrows in both images show two directions where the atomic columns appear aligned.

The significant influence that a small angle tilts can have on the projected images of 2D boron is in stark contrast to the effect on traditional hexagonal 2D vdW materials. **Supplementary Fig. 14** compares simulated STEM BF images of 2D boron (4 layers), and MoS_2 (3 layers) tilted ~2.8° from the electron beam. As observed, the projected image for 2D boron becomes nearly unrecognizable as the (001) plane, while that of MoS_2 still clearly reveals the hexagonal arrangement of its basal plane.

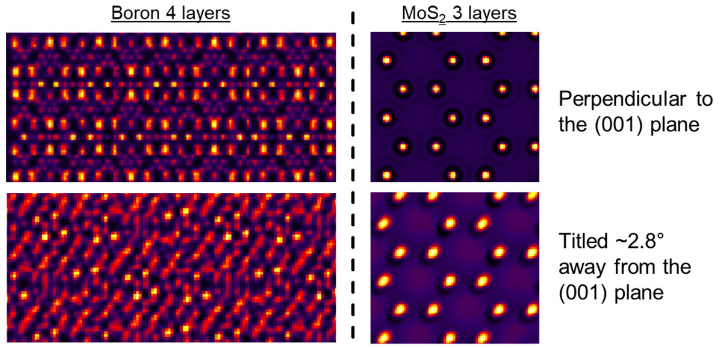

Supplementary Fig. 14. Simulated STEM images of 2D boron (4 layers of the (001) plane), and MoS_2 (3 layers) aligned perpendicular and tilted 2.8° away from the electron beam.

STEM simulation of planar defects in β-rhombohedral boron.

To determine the atomic structure of planar defects from the experimental images, we modelled the defects through Vesta [8] and Atomsk [9], relaxed the models through VASP [10,11], and simulated the models through multislice STEM simulations using the Prismatic code [12]. As shown in **Supplementary Fig. 15**, the simulated images match the experimental data.

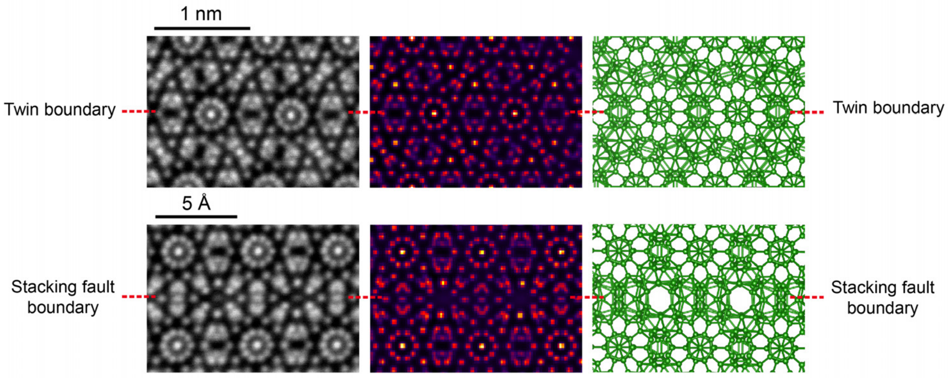

Supplementary Fig. 15. The experimental and simulated STEM images and the corresponding models of the twin boundary (top) and stacking fault boundary (bottom) are shown.

Examples of defects through atomically-resolved STEM

Supplementary Fig. 16 shows the structure of planar defects viewed under different zone-axes, and provide further examples of closely-spaced stacking faults. **Supplementary Fig. 16a** demonstrates twin boundaries from Sample B viewed in the $[1\bar{1}0]$ zone-axis, while **Supplementary Fig. 16b** shows a stacking fault from Sample C viewed under the [110] zone-axis. Quite noticeably for the case of stacking faults, the larger spacing between the (001) planes is its defining feature. In **Supplementary Fig. 16c–d**, two and four layers of the (001) plane bounded by two stacking faults are shown. These examples illustrate that the schematic in **Fig. 5e** in the main text is realistic.

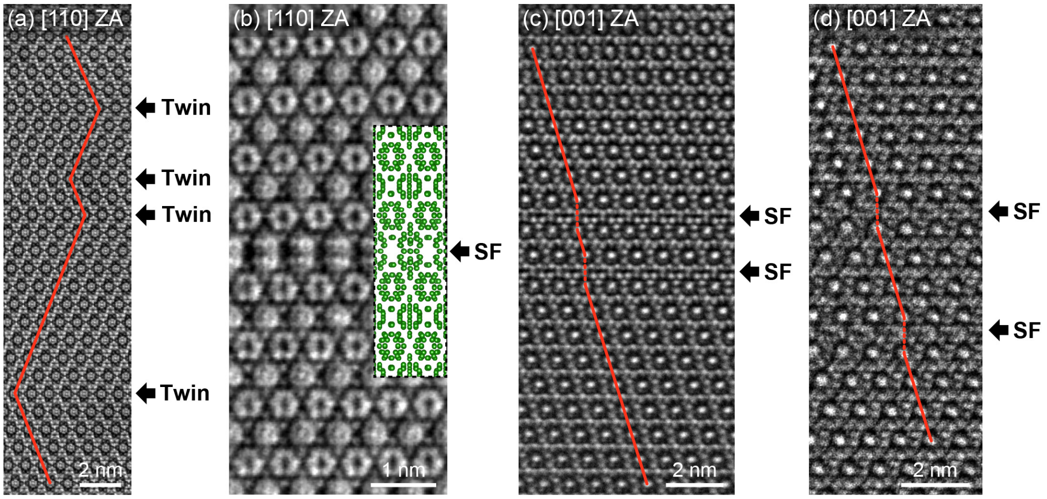

Supplementary Fig. 16. C-BF STEM images of (**a**) twin boundaries in Sample B under the $[1\bar{1}0]$ zone-axis, and (**b**) a stacking fault in Sample C under the [110] zone-axis. (**c**) and (**d**) provide further examples of closely-spaced stacking faults, with the number of (001) planes bounded by the faults to be 2 and 4 layers, respectively.

DFT relaxed models

To evaluate the energy of surface formation from the pristine and faulted boron structures, we followed the approach of Hayami *et al.*, where the surface energies of various planes in α- and β-rhombohedral, and α-tetragonal boron were determined [13]. All structures were relaxed, and the total energies of the bulk pristine and faulted structures were first calculated. A vacuum of ~1 nm was inserted across the twin and stacking fault boundaries (see **Figure 5c** in the main text), and the total energy of both surfaces was recalculated. The difference between the energies of the pristine/faulted structures and the surface structures divided by the surface area is defined as the surface energy, similar to that described by Hayami *et al*. In **Supplementary Fig. 17**, the relaxed models used for the calculations are shown. The red boxes denote the unit cell, which – for visualization of the models – has been duplicated twice in the vertical direction and thrice in the horizontal direction for each model.

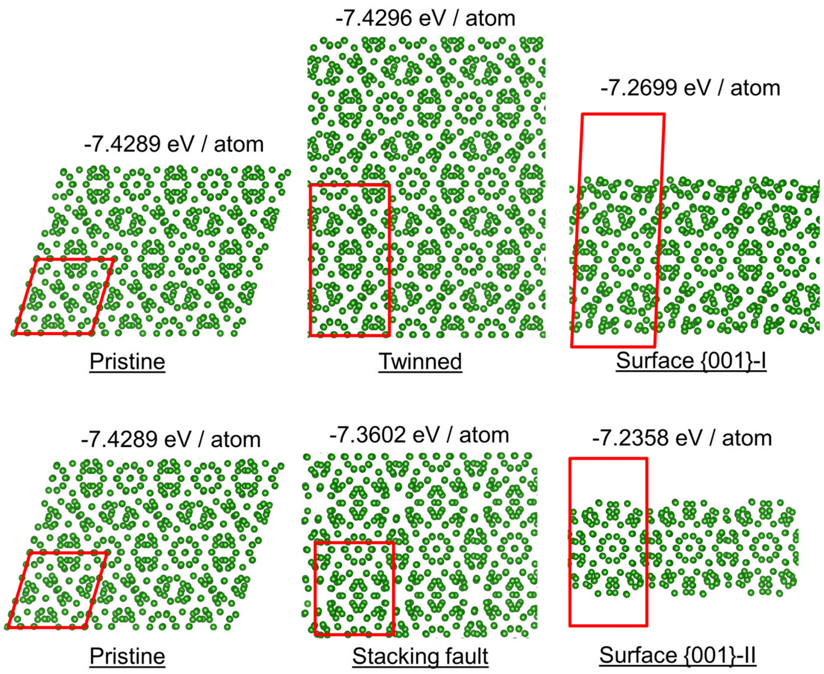

Supplementary Fig. 17. Projected images in the [001] direction of the models used for cleavage energy calculations. The calculated energies per atom are shown above the models. The red box denotes the supercell.

SUPPLEMENTARY REFERENCES

1. Ayache, J., Beaunier, L., Boumendil, J., Ehret, G., & Laub, D. *Sample Preparation Handbook for Transmission Electron Microscopy: Techniques* (Vol. 2). Springer Science & Business Media. (2010).

2. Haar, S., *et al.* Enhancing the Liquid-phase Exfoliation of Graphene in Organic Solvents upon Addition of n-Octylbenzene. *Scientific Reports* **5**, 1-9 (2015).

3. Li, H. *et al.* Scalable Production of Few-Layer Boron Sheets by Liquid-Phase Exfoliation and Their Superior Supercapacitive Performance. *ACS Nano* **12**, 1262–1272 (2018).

4. Feng, B. *et al.* Experimental realization of two-dimensional boron sheets. *Nature Chemistry.* **8**, 563–568 (2016).

5. Kaur, H. *et al.* Production of Quasi-2D Platelets of Nonlayered Iron Pyrite (FeS2) by Liquid-Phase Exfoliation for High Performance Battery Electrodes. *ACS Nano* **14**, 13418-13432 (2020).

6. Liu, G. *et al.* Visible-light-responsive β-rhombohedral boron photocatalysts. *Angewandte Chemie International Edition* **52**, 6242-6245 (2013).

7. Fan, Q. *et al.* High-yield production of few-layer boron nanosheets for efficient electrocatalytic N_2 reduction. *Chem. Commun.* **55**, 4246–4249 (2019).

8. Momma, K. & Izumi, F. VESTA 3 for Three-Dimensional Visualization of Crystal, Volumetric and Morphology Data. *Journal of Applied Crystallography* **44**, 1272-1276 (2011).

9. Hirel, P. Atomsk: A Tool for Manipulating and Converting Atomic Data Files. *Computer Physics Communications* **197**, 212-219 (2015).

10. Kresse, G. & Furthmüller, J. Efficient Iterative Schemes for *ab initio* Total-Energy Calculations Using a Plane-Wave Basis Set. *Physical Review B* **54**, 11169 (1996).

11. Kresse, G. & Joubert, D. From Ultrasoft Pseudopotentials to the Projector Augmented-Wave Method. *Physical Review B* **59**, 1758 (1999).

12. DaCosta, L. R. *et al.* Prismatic 2.0 – Simulation Software for Scanning and High Resolution Transmission Electron Microscopy (STEM and HRTEM). *Micron* **151**, 103141 (2021).

13. Hayami, W. & Otani, S. The Role of Surface Energy in the Growth of Boron Crystals. *The Journal of Physical Chemistry C* **111**, 688-692 (2007).
